# Coral calcification under daily oxygen saturation and pH dynamics reveals the important role of oxygen

**DOI:** 10.1242/bio.20147922

**Published:** 2014-05-23

**Authors:** Tim Wijgerde, Catarina I. F. Silva, Vera Scherders, Judith van Bleijswijk, Ronald Osinga

**Affiliations:** 1 Aquaculture and Fisheries Group, Department of Animal Sciences, Wageningen University, Wageningen University and Research Centre, 6709 PG Wageningen, The Netherlands; 2 Biological Oceanography, Royal Netherlands Institute for Sea Research, 1797 SZ 't Horntje, The Netherlands

**Keywords:** *Acropora millepora*, Calcification, Climate change, Oxygen

## Abstract

Coral reefs are essential to many nations, and are currently in global decline. Although climate models predict decreases in seawater pH (∼0.3 units) and oxygen saturation (∼5 percentage points), these are exceeded by the current daily pH and oxygen fluctuations on many reefs (pH 7.8–8.7 and 27–241% O_2_ saturation). We investigated the effect of oxygen and pH fluctuations on coral calcification in the laboratory using the model species *Acropora millepora*. Light calcification rates were greatly enhanced (+178%) by increased seawater pH, but only at normoxia; hyperoxia completely negated this positive effect. Dark calcification rates were significantly inhibited (51–75%) at hypoxia, whereas pH had no effect. Our preliminary results suggest that within the current oxygen and pH range, oxygen has substantial control over coral growth, whereas the role of pH is limited. This has implications for reef formation in this era of rapid climate change, which is accompanied by a decrease in seawater oxygen saturation owing to higher water temperatures and coastal eutrophication.

## INTRODUCTION

Coral reefs provide essential goods and ecosystem services to many nations worldwide (Moberg and Folke, 1999). Unfortunately, coral reefs are currently in severe global decline due to climate change, ocean acidification and local anthropogenic disturbances such as coastal development, overfishing and pollution (Hughes et al., 2003; Mora et al., 2013). A recent study on reefs growing near submarine CO_2_ vents showed that a predicted global oceanic pH of 7.8 at the year 2100 may lead to dramatic changes in reef ecosystems, with severely reduced biodiversity, recruitment and abundance of complex reef builders (Fabricius et al., 2011). Interestingly, the projected pH decrease for this century is greatly exceeded by the daily pH fluctuation to which Indo-Pacific corals on reef flats and lagoons are exposed, which ranges from 8.7 during the day to 7.8 at night (Ohde and van Woesik, 1999). In addition, the seawater oxygen saturation at these reef locales shows dramatic changes over a diel cycle, with a range of 241% air saturation (or 14.19 mg O_2_ L^−1^) at daytime, to 27% saturation (or 1.67 mg O_2_ L^−1^) at night (Ohde and van Woesik, 1999).

A change in seawater pH will affect the carbonate ion concentration and aragonite saturation state of seawater, which all are correlated with coral calcification rates (Marubini et al., 2003; Schneider and Erez, 2006). The proton flux hypothesis suggests that these correlations are mainly caused by changes in proton efflux rates from coral tissue at different pH values, affecting the calcification reaction in the calicoblastic medium (Jokiel, 2011; Jokiel, 2013). Changes in the oxygen saturation of seawater are also known to influence coral calcification rates (Rinkevich and Loya, 1984; Al-Horani et al., 2007; Colombo-Pallotta et al., 2010; Wijgerde et al., 2012), possibly because oxygen is an important substrate for ATP synthesis (Babcock and Wikström, 1992), which in turn is required for the energy-driven process of calcification (Chalker and Taylor, 1975; Ip et al., 1991; Zoccola et al., 2004). To further complicate matters, coral calcification rates may be enhanced by light, a phenomenon known as light-enhanced calcification (Kawaguti and Sakumoto, 1948; Chalker and Taylor, 1975). This enhancement of calcification by light may be explained by photosynthesis of endosymbiotic zooxanthellae, providing energy for calcification (Kühl et al., 1995; Colombo-Pallotta et al., 2010), while simultaneously increasing the pH of the polyp coelenteron and calicoblastic medium, stimulating calcium carbonate deposition (Furla et al., 2000; Al-Horani et al., 2003; Venn et al., 2011). Thus, the ability of corals to produce oxygen and upregulate internal pH via photosynthesis may result in a different calcification response to changes in the external environment in light when compared to darkness.

Studying how corals respond to extreme fluctuations in pH and oxygen saturation will increase our understanding of reef formation in this highly dynamic environment. In addition, these findings will provide insight into how corals will respond to future climate change, which is accompanied by decreased pH owing to absorption of atmospheric CO_2_ by seawater (Mora et al., 2013) and decreased seawater oxygen saturation, due to higher water temperatures and coastal eutrophication (Vaquer-Sunyer and Duarte, 2008; Cheung et al., 2013; Mora et al., 2013).

We investigated how natural oxygen and pH fluctuations affect light and dark calcification of corals using the species *Acropora millepora*, a major reef-builder in the Indo-Pacific. By using factorial designs, we experimentally disentangled the individual roles played by oxygen and pH in coral growth. In a first experiment, we measured dark calcification of corals exposed to normoxia (100% saturation) and hypoxia (30% saturation), at both a pH of 8.1 and 7.8. During a follow-up experiment, light calcification rates were measured at normoxia (100%) and hyperoxia (170%), at a pH of 8.1 and 8.4. These specific treatments were chosen as they represent actual fluctuations known to occur on coral reefs (Ohde and van Woesik, 1999).

## MATERIALS AND METHODS

### Coral fragmentation and husbandry

For this study, we used the Indo-Pacific scleractinian species *Acropora millepora* (Ehrenberg 1834), a colony of which was obtained from a commercial supplier (De Jong Marinelife BV, Spijk, The Netherlands). This colony had been in aquaculture for approximately two years at the onset of the experiments described here. Coral fragments (*n* = 32) were randomly cut from a single colony, and vertically glued onto 5×5 cm PVC tiles (Wageningen UR, Wageningen, The Netherlands) using cyanoacrylate (Gamma BV, Wageningen, The Netherlands). Branches were cut evenly, resulting in uniformly bifurcated fragments 3–4 cm in height. All fragments were allowed to recover for three weeks in a 400 L holding aquarium.

The holding aquarium was provided with full spectrum white light, at an quantum irradiance (QI) of 230 µmol m^−2^ s^−1^ (12 h:12 h light:dark regime), created by two 4×54 W T5 fixtures (Elke Müller Aquarientechnik, Hamm, Germany). Water flow was provided by one Turbelle nanostream 6085 circulation pump (Tunze Aquarientechnik GmbH, Penzberg, Germany) providing a total flow rate of 8,000 L h^−1^. Water parameters were maintained at the following levels (means ± S.D.): salinity 35.0±0.1 g L^−1^, temperature 26±0.3°C, pH 8.2±0.2, ammonium 0.01±0.01 mg L^−1^, nitrate 0.13±0.03 mg L^−1^, phosphate 0.02±0.01 mg L^−1^, calcium 400±15 mg L^−1^, magnesium 1300±45 mg L^−1^.

### Calcification measurements

To measure calcification rates, we used the alkalinity anomaly technique as previously described (Wijgerde et al., 2012). Colonies (*n* = 4 per treatment, *n* = 32 in total, where each coral was only used once) were subjected to a total of 8 different treatments (4 light and 4 dark treatments) in 2 randomised factorial designs. The light and dark experiments were each carried out over a one-week period, with an interlude of several weeks between the two experiments. Under light conditions (QI of 230 µmol m^−2^ s^−1^), corals were exposed to ambient oxygen saturations of 100 or 170% (6.36 or 10.81 mg O_2_ L^−1^, respectively), at both a pH of 8.1 and 8.4, resulting in 4 treatments in total. We chose to maintain the same QI as in the holding aquarium, to prevent light-induced stress, which may confound the results. In addition, scleractinian corals that are acclimated and subsequently exposed to a QI of at least up to 300 µmol m^−2^ s^−1^ show saturation of photosynthesis (Osinga et al., 2011). Under dark conditions, corals were exposed to oxygen saturations of 100 or 30% (6.36 or 1.91 mg O_2_ L^−1^, respectively), at both a pH of 8.1 and 7.8, again yielding 4 treatments in total. We specifically chose these treatments to compare the effects of natural day–night oxygen and pH fluctuations found on coral reefs. To avoid pseudoreplication, each coral colony was incubated for 5 hours in a separate cell with a net water volume of 1250 ml. All corals were acclimated to each experimental condition for 30 minutes before the start of every experiment. To maintain stable oxygen saturations during the entire incubations, five 5850S smart flow mass controllers (Brooks International, Hatfield, USA) were connected to two digital microprocessor units, models 0152/0154 (Brooks International, Hatfield, USA), which allowed for controlling volumetric flow rates of gases in each cell. Nitrogen (N_2_) and oxygen (O_2_) gas were used for the hypoxia and hyperoxia treatments, respectively. Compressed air was used for the normoxia treatment. Oxygen concentrations were monitored throughout all experiments with IntelliCAL^TM^ LDO101 luminescent dissolved oxygen probes (Hach-Lange GmbH, Düsseldorf, Germany). The pH was measured and controlled with CO_2_ computers and calibrated pH sensors (Resun, Longgang, China), which controlled addition of CO_2_ via solenoid valves (AquaHolland, Dordrecht, The Netherlands). Each pH sensor was calibrated before every experiment using pH 7 and pH 10 buffers (WTW GmbH, Weilheim, Germany) enriched in 25.4 g L^−1^ NaCl to approximate the ionic strength of seawater. Temperature was kept at 26°C with water jackets surrounding each incubation cell, which were connected to a water bath equipped with a TC20 water cooler (Teco SRL, Ravenna, Italy). Oxygen, pH and temperature values of all treatments were highly stable during the incubation periods (Table 2). Water flow was provided with magnetic stirring plates (IKA Werke GmbH and Co. KG, Staufen, Germany) and was estimated at approximately 5 cm s^−1^. Water from the maintenance system was used to fill the incubation chambers, to minimise stress to the coral colonies. Calcium and alkalinity were always measured and adjusted when required to 400 mg L^−1^ and 2.40 mEq L^−1^ (or 2,346 µmol kg^−1^), respectively, before every experiment. A water sample of 50 mL each was taken from every incubation chamber at t = 0 (defined as the time point directly following the acclimation period) and t = 5 hours for determination of total alkalinity (A_T_). This was taken into account during calculation of net cell water volumes. To determine A_T_, 40 ml samples were potentiometrically titrated on a Titralab 840 (Radiometer Analytical SAS, Lyon, France) with 0.02 M HCl to inflection point. Changes in A_T_, expressed in mEq L^−1^, were calculated for each cell. During each experiment, a control cell containing only the same seawater was used, and background alkalinity changes were used to correct all data. Total alkalinity depletions in mEq were calculated by taking net cell volumes into account. These were subsequently converted to mg calcium carbonate (CaCO_3_) produced by the corals, by using a mEq to mg CaCO_3_ ratio of 1:50.04. Differences in coral biomass were taken into account by using the buoyant weight of each coral, corrected for combined PVC tile and glue weight, obtained before each experiment. This method was chosen as it is highly accurate and valid as branching corals cultured under similar conditions have a constant surface/volume and surface/mass ratio (Osinga et al., 2011). All data were expressed as mg CaCO_3_ per gram coral per hour. To minimise the potential effect of time of day, all experiments were conducted within the same time interval of 9:00 to 17:00 hrs.

### Data analysis

Normality of data was evaluated by plotting residuals of each dataset versus predicted values, and by performing a Shapiro–Wilk test. Homogeneity of variances was determined with Levene's test. All data were found to be normally distributed and showed homogeneity of variances (*P*>0.050). We used a two-way factorial ANOVA to determine the main and interactive effects of oxygen and pH on light and dark calcification rates of *Acropora millepora*. Simple effect contrasts were used to elucidate interactive effects. Statistical analysis was performed with IBM SPSS Statistics 22 (IBM Corp., Armonk, USA). Graphs were plotted with SigmaPlot 11.0 (Systat Software, Inc., San Jose, USA). Data presented are means + standard error (S.E.) or means ± standard deviation (S.D.), as indicated.

## RESULTS

**Fig. 1.**
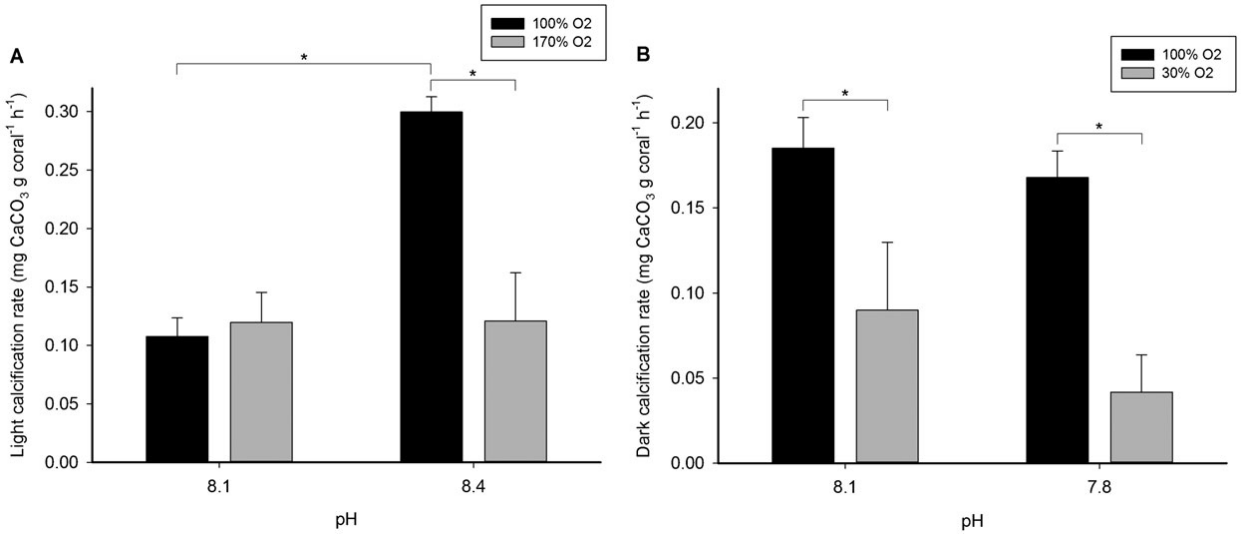
Calcification rates of *Acropora millepora*. (A) Light calcification rate of *A. millepora* at a pH of 8.1 and 8.4, and 100 and 170% oxygen saturation. (B) Dark calcification rate of *A. millepora* at a pH of 8.1 and 7.8, and 100 and 30% oxygen saturation. Values are means + S.E. (*n* = 4). Asterisks indicate significant differences (*P*<0.050). The light and dark experiments were carried out with an interlude of several weeks.

Light calcification rates of the scleractinian coral *Acropora millepora* were variable between treatments and ranged from 0.11±0.02 to 0.30±0.01 mg CaCO_3_ g coral^−1^ h^−1^ (Fig. 1). Significant main and interactive effects of oxygen and pH on light calcification rates were found (Table 1). The interaction was reflected by a significant effect of oxygen at a pH of 8.4 only (F_1,12_ = 22.991, *P* = 0.000). At pH 8.4, calcification rates were 60% lower at hyperoxia (170% oxygen saturation) when compared to normoxia (100% saturation) (*P* = 0.000). At pH 8.1, no significant difference in calcification rates between 100% and 170% oxygen saturation was found (*P* = 0.749). Conversely, the interaction was due to a significant effect of pH at an oxygen saturation of 100% only (F_1,12_ = 26.498, *P* = 0.000). At 100% oxygen saturation, calcification rates were 178% higher at a pH of 8.4 as compared to 8.1 (*P* = 0.000). At 170% oxygen saturation, no significant difference in calcification rates between pH 8.1 and 8.4 was found (*P* = 0.980).

**Table 1.**
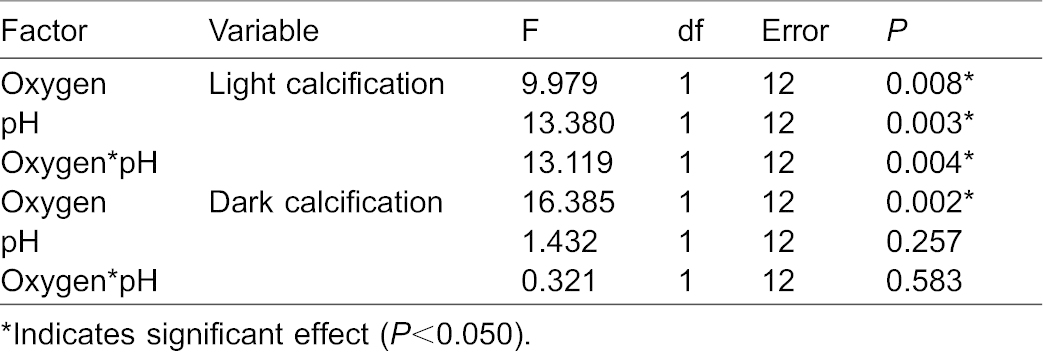
Two-way factorial ANOVA, demonstrating main and interactive effects of oxygen and pH on light and dark calcification rates of *Acropora millepora* (*n* = 4)

**Table 2.**
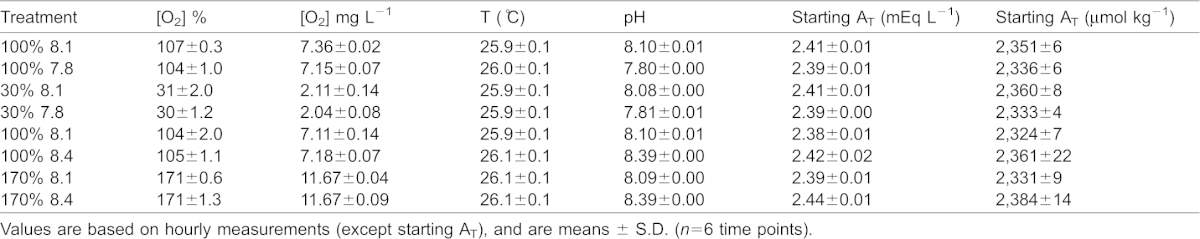
Oxygen, pH, temperature and starting A_T_ (*n* = 5 cells) of each experimental condition

Dark calcification rates of *A. millepora* ranged from 0.04±0.02 to 0.19±0.02 (Fig. 1). A significant main effect of oxygen on dark calcification rates was found (Table 1), with a 50–75% decrease in calcification rates at hypoxia (30% oxygen saturation) as compared to normoxia (100% saturation), irrespective of pH level (*P* = 0.037 and *P* = 0.006 at a pH of 8.1 and 7.8, respectively). No (interactive) effect of pH on dark calcification rates was found (Table 1).

## DISCUSSION

The results clearly show the profound effects of seawater oxygen saturation on light and dark calcification rates of *A. millepora*, whereas the role of pH in our experiments was limited to light conditions. The calcification rates we observed (0.11±0.02 to 0.30±0.01 mg CaCO_3_ g coral^−1^ h^−1^, or 2.59±0.38 to 7.20±0.31 mg CaCO_3_ g coral^−1^ day^−1^) are comparable to previously reported growth rates of several scleractinian corals, including *Galaxea fascicularis*, *Acropora verweyi* and *Oculina arbuscula* (Marubini et al., 2003; Ries et al., 2009). Our findings are in line with the recently documented positive linear relationship between oxygen saturation and calcification rates of the scleractinian coral *Galaxea fascicularis* (Wijgerde et al., 2012), and the enhancing effects of seawater aeration on dark calcification rates of *Montastraea faveolata* (Colombo-Pallotta et al., 2010) and *Stylophora pistillata* (Rinkevich and Loya, 1984). The limiting role of oxygen in dark calcification rates is probably caused by decreased ATP synthesis in calicoblastic cells via aerobic respiration, resulting in less available energy for calcification (Chalker and Taylor, 1975; Ip et al., 1991).

Although an elevated pH had a positive effect on light calcification rates of *A. millepora*, this beneficial effect was completely negated by hyperoxia. A possible explanation for this phenomenon is oxygen intoxication due to reduced oxygen release by the coral (Kühl et al., 1995; Mass et al., 2010), resulting in cellular damage (Wijgerde et al., 2012; and references therein) and photorespiration (Mass et al., 2010). This may coax corals into investing metabolic energy in the production of anti-oxidants rather than calcification. At higher water flow rates, this negative effect of hyperoxia on light calcification rates may be less prominent due to flow-enhanced mass transfer of oxygen (Mass et al., 2010; Chindapol et al., 2013). As an elevated pH of coral reef waters during daytime is always accompanied by hyperoxia (Ohde and van Woesik, 1999), most likely due to photosynthesis of benthic organisms, this suggests that increased pH has no significant effect on *in situ* light calcification rates of *A. millepora*. Similarly, within the naturally observed range, pH had no significant effect on dark calcification rates. Although several studies have shown that a pH decrease from 8.1 to 7.8 results in decreased calcification rates of certain coral species (e.g. Fabricius et al., 2011), our findings may be specific to *A. millepora*. It is also possible that our results are genotype-specific, as different genotypes within a given species are known to display growth variation in aquaria (Osinga et al., 2011). Although long-term exposure (i.e. weeks to months) to decreased seawater pH may reveal a significant negative effect on this species, our short-term incubations are highly relevant to the dynamic *in situ* conditions on coral reefs, with the oxygen saturation and pH fluctuating on a time scale of hours (Ohde and van Woesik, 1999).

Interestingly, we did not find evidence for the theory of light-enhanced calcification (Kawaguti and Sakumoto, 1948; Chalker and Taylor, 1975) at baseline conditions of pH 8.1 and normoxia, as light calcification rates were lower than dark calcification rates. This observation is in agreement with a recent study on the growth of *Galaxea fascicularis*, during which no significant difference between light and dark calcification rates was found when the incubation water was aerated to maintain normoxia (Wijgerde et al., 2012), although it contrasts with several previous studies (reviewed by Gattuso et al., 1999). It seems that light-enhanced calcification is only found when dark calcification rates are impaired due to oxygen limitation and/or reduced oxygen mass transfer at low water flow. It must be noted, however, that the light and dark experiments were carried out with an interlude of several weeks. It is therefore possible that other time-dependent factors in the holding aquarium were responsible for the observed differences between the light and dark experiments.

In conclusion, our results suggest that seawater oxygen saturation is a major determinant of day–night coral calcification rates, whereas the role of pH is limited within the applied oxygen range, at least for the major reef-builder *Acropora millepora*. These findings are in line with the documented positive effects of seawater oxygenation on calcification rates of *G. fascicularis*, *M. faveolata* and *S. pistillata* (Rinkevich and Loya, 1984; Colombo-Pallotta et al., 2010; Wijgerde et al., 2012). Our results have implications for coral reef formation in this era of rapid climate change, which is accompanied by a decrease in seawater oxygen saturation owing to higher water temperatures and coastal eutrophication (Vaquer-Sunyer and Duarte, 2008; Cheung et al., 2013; Mora et al., 2013). As our conclusions are based on preliminary laboratory experiments, future *in situ* studies should determine to what extent daily oxygen profiles correlate with reef calcification rates, and how different coral species react to different combinations of dissolved oxygen and pH. Disentangling the confounding effects of pH, oxygen and temperature fluctuations *in situ* will be a challenge, however.
